# TINCR expression is associated with unfavorable prognosis in patients with hepatocellular carcinoma

**DOI:** 10.1042/BSR20170301

**Published:** 2017-07-27

**Authors:** Feng Tian, Jian Xu, Fangxi Xue, Encui Guan, Xiaoguang Xu

**Affiliations:** Department of Gastroenterology, Yishui Central Hospital of Linyi City, Yishui 276400, Shandong Province, P.R. China

**Keywords:** disease-free survival (DFS), hepatocellular carcinoma (HCC), long noncoding RNAs (lncRNAs), overall survival (OS), prognosis, TINCR

## Abstract

Emerging evidence are accumulating that long noncoding RNAs (lncRNAs) have recently been identified to participate in various cellular processes. Terminal differentiation induced ncRNA (TINCR) is a newly identified lncRNA with its functional roles not fully elucidated in human malignancy. The current study aims to identify the clinical significance of TINCR in prognosis and malignant progression of hepatocellular carcinoma (HCC). TINCR expression in HCC specimens at various stages of tumorigenesis were measured by quantitative real-time RT PCR (qRT-PCR). The matched para-carcinoma tissues were used as controls. The associations of TINCR with clinicopathological characteristics, disease-free survival (DFS) and overall survival (OS) of patients were further evaluated. Results revealed that high TINCR expression was significantly correlated with tumor size (*P*=0.005), tumor differentiation status (*P*=0.017), TNM stage (*P*=0.010), and vascular invasion (*P*=0.004). Moreover, Kaplan–Meier analysis demonstrated that TINCR was correlated to both DFS and OS in HCC cohorts. Patients with high TINCR expression tended to have worse prognosis. Multivariate Cox regression analysis indicated that TINCR was an independent poor prognostic indicator for DFS (HR =1.32, 95% CI: 1.00–1.57, *P*=0.000) and OS (HR =1.57, 95% CI: 1.30–1.86, *P*=0.004) in HCC. TINCR was demonstrated as a direct target of *miR-137* and *miR-133a*, and was suppressed by *miR-137*/*miR-133a*. These results provide the first evidence that the expression of TINCR in HCC may play an oncogenic role in HCC differentiation, invasion, and metastasis. *miR-137*/*miR-133a*-TINCR pathway may serve as a promising target for tumor recurrence and prognosis of patients with HCC.

## Introduction

Next generation sequencing technology has revealed that above 90% of the human genome is transcribed to generate an extraordinary range of nonprotein-coding (noncoding) RNAs [1,2]. Long noncoding RNAs (lncRNAs) have no protein-coding function, which are greater than 200 nts in length, influential in remarkable biological processes [3,4]. Compared with miRNAs, lncRNAs are less well understood despite recent interest and attention [5]. Multiple studies have manifested that lncRNAs are involved in wide ranges of physiological and pathophysiological processes, acting in negative or positive feedback loops as oncogenes or tumor suppressor genes [6]. They have been validated as powerful regulatory factors contributing to various cellular processes, including chromatin modifications, gene imprinting, alternative splicing, genome rearrangement, cell proliferation, migration, apoptosis, as well as nuclear–cytoplasmic trafficking [7–9]. In addition, it reported that lncRNAs could be served as promising biomarkers for diagnosis and potential therapeutic targets in cancers [10]. Therefore, lncRNAs might perform extensive and complex functions in carcinogenesis and human malignancy progression [11]. There are also evidence suggesting that a panel of lncRNAs have been implicated to have abnormal expression patterns in various cancers, and were associated with tumor cell proliferation, apoptosis, metastasis, and invasion [12–14].

  Terminal differentiation induced ncRNA (TINCR), which was an uncharacterized lncRNA, was demonstrated to be one of the most highly induced lncRNAs of the annotated noncoding RNAs (ncRNAs) during differentiation. The gene of TINCR resides on chromosome 19 in human genome between the *SAFB2* and *ZNRF4* genes [15]. Since that, the prognostic implication of TINCR was rarely studied in hepatocellular carcinoma (HCC). To better understand the functional impact of TINCR, we investigated the TINCR expression level in clinical HCC specimens and adjacent normal control tissues, as well as analyzed its association with disease-free survival (DFS) and overall survival (OS) of patients.

## Materials and methods

### Patients and specimens

The current study has been approved by the Ethics Committee of the Second Affiliated Hospital of Henan University of Chinese Medicine. All patients involved in the present study have provided written informed consents. Briefly, fresh clinical HCC specimens and the adjacent normal tissues were collected from 248 patients who underwent surgery between January 2008 and September 2009 in the Second Affiliated Hospital of Henan University of Chinese Medicine. Tumor TNM stages were identified according to the American Joint Committee on Cancer 2010 TNM classification. All the fresh tissues were put into liquid nitrogen post-operation. The tissues were then stored into a −80°C refrigerator for use. Exclusion criteria: patients receiving any chemotherapy or radiotherapy prior to the surgery or with other cancers. The histopathology of all the tissue specimens were confirmed double-blind by two pathologists in Department of Gastroenterology, Yishui Central Hospital of Linyi City.

### Measurement of end points

DFS is defined as the time interval elapsed from surgery to the first occurrence of any of the following events: recurrence of HCC, HCC distant metastasis, or death from any cause without documentation of a cancer-related event. OS is defined as the time interval elapsed from surgery to death in patients with HCC. Death of participants was ascertained by reporting from the family and verified by review of public record. DFS and OS status was recorded by clinical staff blinded to participant status, including clinicopathologic or TINCR expression data.

### Cell culture

HUH7 (human HCC cell line) were purchased from the American Type Culture Collection (Manassas, VA, U.S.A.). The cells were maintained in RPMI-1640 medium (Cat# 11354-035, Invitrogen-Gibco) containing 10% heat-inactivated FBS (Cat# 0354-4213, Invitrogen-Gibco), 2 mM L-glutamine, and 100 U/ml penicillin/streptomycin at 37°C in a humidified atmosphere containing 5% CO_2_. For cell transfection, exponentially growing cells (1.5 × 10^5^) were seeded in 12-well plates and transfected with 30 nM miRNA mimics or the negative control (GenePharma, Shanghai, China) using Lipofectamine 3000 (Life Technologies, Carlsbad, CA, U.S.A.) according to the manufacturer’s instructions.

### Quantitative real-time RT-PCR (qRT-PCR)

Total RNA was extracted from clinical specimens using the TRIzol reagent (Cat# 15596-026, Gibco BRL and Life Technologies) following manufacturer’s instructions. The total RNA was reverse transcribed to cDNA using a cDNA Reverse Transcription Kit (Cat# 35412-011, Promega Inc., U.S.A.). cDNA sample was analyzed in triplicate on an ABI 7500 system (Applied Biosystems) using SYBR Green according to the manufacturer’s instructions. ACTIN was used as an internal control. Primers used were as follows: TINCR, forward: 5′-CCATCCCTCTGTAACCACCT-3′ and reverse: 5′-GTTGGGTCTAGATTCCAGCA-3′; ACTIN, forward: 5′-ATCATGTTTGCCTAGATCAACA-3′ and reverse: 5′-CATCTCTTGCTAAGCGTCCA-3′. The PCR conditions were as follows: 95°C for 10 min for initial denaturation followed by 45 cycles of 95°C for 10 s, 60°C for 35 s, 72°C for 30 s, and then a final extension of 10 min. The relative mRNA levels were quantitated and analyzed using the SDS system software (Applied Biosystems) The relative quantitation of *TINCR* mRNA was performed using the 2^−ΔΔ*C*^_t_ method. The experiments were repeated at least thrice.

### Western blotting

Cells were washed and lysed in lysis buffer (10 mM KCl, 20 mM HEPES, 5 mM EDTA, 1% NP-40, 0.25% deoxycholate, pH 7.4) containing protease and phosphatase inhibitors. After incubation on ice for 20 min, the lysate was centrifuged at 12000 r.p.m. for 20 min at 4°C, and the supernatant was collected. The protein concentration was assessed using a standard BCA assay (Beyotime). For each sample, 60 μg of protein was separated on SDS-PAGE (10% gel) (Bio–Rad), transferred on to a PVDF membrane (Cat#: 3010040001; Roche Applied Science, Mannheim, Germany). After blocking with 5% milk in TBST (0.1% TBS-Tween 20) at room temperature for 1.5 h, the membrane was washed three times with TBST and incubated with monoclonal mouse anti-TINCR polyclonal antibody (1:2000, Abcam, Cambridge, MA, U.S.A.) or monoclonal rabbit anti-β-actin antibody (1:2000, Abcam, Cambridge, MA, U.S.A.) overnight at 4°C. After washing with PBS for 10 min, the membrane was incubated with mouse anti-rabbit secondary antibody (1:20000, Abcam, Cambridge, MA, U.S.A.) for 1 h at room temperature. After washing with PBS for 15 min, protein bands were detected using the ECL Western Blotting Kit (Pierce Chemical, Rockford, IL, U.S.A.). The relative protein expression was analyzed by Image-Pro Plus software 6.0, represented as the density ratio compared with β-actin.

### Luciferase reporter assay

The *TINCR* mRNA 3′-UTR fragment was amplified by PCR and inserted into the pmirGLO miRNA target expression vector (Promega, San Luis Obispo, CA, U.S.A.) to construct pmirGLO-TINCR (3′-UTR) plasmid. For the luciferase reporter assay, cells were cotransfected with miRNA mimics (50 nM) or negative control (50 nM), and pmirGLO-TINCR (3′-UTR) (50 nM) with Lipofectamine 3000 (Life Technologies, Carlsbad, CA, U.S.A.), according to the manufacturer’s instructions. After transfection for 48 h, the activities of *Renilla* luciferase and firefly luciferase were determined by the dual-luciferase reporter assay system (Promega). The luciferase activity was normalized to the firefly luciferase activity.

### Statistical analysis

Statistical analysis was performed using the R Statistical Software Package (version 3.1.3). Statistical comparisons were performed using Student’s *t* test. The correlations between TINCR expression level and the clinical parameters were analyzed by Pearson’s χ^2^ test or Fisher’s exact test. DFS and OS survival curves were analyzed by the Kaplan–Meier method, and the difference was calculated by the log rank test. The independence of TINCR in prognosis was estimated using the multivariate Cox proportional hazards regression model. Differences were considered significant for *P*<0.05.

## Results

### TINCR expression in HCC patients

**Figure 1:**
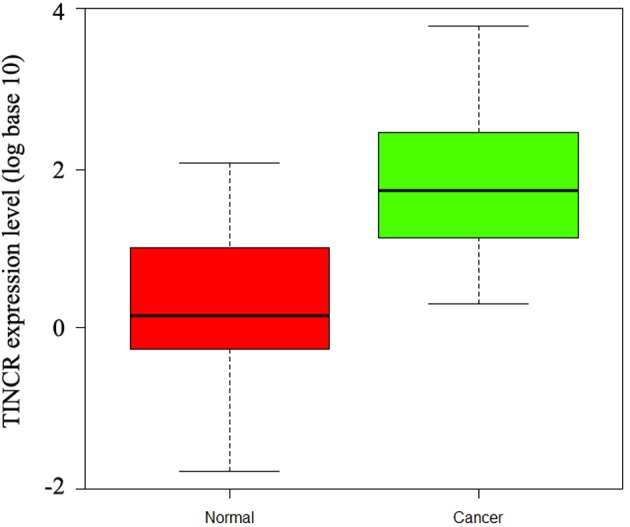
Expression of TINCR detected by real-time PCR Relative expression of TINCR in HCC specimens and adjacent normal control liver tissues (*n*=248).

The expression levels of TINCR in 248 HCC samples and matched para-carcinoma specimens were detected by quantitative real-time RT PCR (qRT-PCR). Results showed that *TINCR* mRNA expression level was significantly up-regulated in HCC samples compared with the corresponding control samples (P<0.01, Figure 1), suggesting that aberrant TINCR expression might be correlated with HCC pathogenesis. Then, we assigned HCC samples to TINCR low-expression group and TINCR high-expression group that were tumors with TINCR expression under and above the median value of expression in all 248 gliomas, respectively (*n*=138 and 110 for low-expression group and high-expression group, respectively).

### Association of TINCR expression with clinicopathological parameters of HCC patients

After tumor tissues were classified into two subgroups (high-TINCR expression subgroup and low-TINCR expression subgroup), the association of TINCR expression level with clinicopathological characteristics of HCC patients were further analyzed. Results showed that the TINCR expression level was associated with tumor size (*P*=0.005), differentiation (*P*=0.017), vascular invasion (*P*=0.004), and TNM stage (*P*=0.010), which indicated that high TINCR expression was probably to present larger tumor size, advanced clinical stage, enhanced invasion degree, and less tumor differentiation. However, no correlation was found between TINCR expression and gender, age at diagnosis, alanine transaminase (ALT), α-fetoprotein (AFP), hepatitis B surface antigen (HbsAg), or hepatitis history (*P*>0.05, Table 1).

**Table 1:** Association of TINCR expression with clinicopathologic parameters

Clinicopathologic parameters	*n*	TINCR expression		*P*-value
		Low	High	
Total	248	138	110	
**Age**				0.194
<55 years	133	74	59	
≥55 years	115	64	51	
**Gender**				0.764
Male	184	103	81	
Female	64	35	29	
**ALT (U/l)**				0.851
≤75	211	116	95	
>75	37	22	15	
**AFP (ng/ml)**				0.307
>20	157	81	76	
≤20	91	57	34	
**Hepatitis history**				0.772
Present	179	101	78	
Absent	69	37	32	
**Vascular invasion**				0.004
Present	69	29	40	
Absent	179	109	70	
**Liver cirrhosis**				0.861
Present	122	61	61	
Absent	126	77	49	
**Tumor size (cm)**				0.005
≤5	124	77	47	
>5	124	61	63	
**Differentiation status**				0.017
Well/Moderate	165	100	65	
Poor-undifferentiated	83	38	45	
**TNM stage**				0.010
I + II	159	97	62	
III + IV	89	41	48	
**HbsAg**				0.881
Present	44	23	21	
Absent	204	115	89	

### Association of TINCR expression with DFS of patients

**Figure 2:**
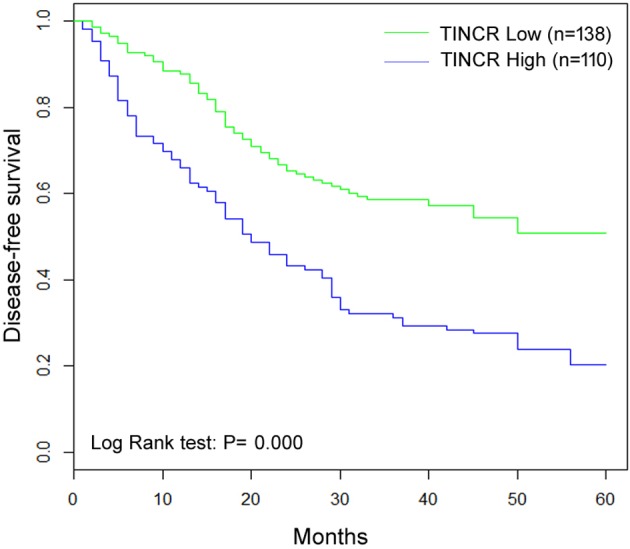
Kaplan–Meier curves for DFS of HCC patients with high and low *TINCR* mRNA expression levels Patients with high *TINCR* mRNA levels (fold change ≥2) tended to have a shorter DFS time (*P*=0.000).

The association of DFS and TINCR expression of HCC patients was evaluated by Kaplan–Meier analysis (Table 2). Results showed that patients with high TINCR expression in HCC tissues had unfavorable DFS compared to those with low TINCR expression (Figure 2, log-rank test: P=0.017). At the 5-year follow-up, disease-free patients with low TINCR expression accounted for 47.5%, whereas disease-free patients with high TINCR expression accounted for 22.3%. This result indicated that HCC patients with high TINCR expression had a relatively higher risk of tumor relapse compared to those with low TINCR expression. Also, tumor size (log-rank test: *P*=0.001), AFP (log-rank test: *P*=0.002), differentiation status (log-rank test: *P*=0.036) and TNM stage (log-rank test: *P*=0.004), and vascular invasion (log-rank test: *P*=0.000) were also shown to be associated with DFS. However, no significant correlation was found amongst gender, age at diagnosis, or liver cirrhosis and DFS of patients with HCC. Moreover, multivariate analysis showed that TINCR as an independent prognostic factor for DFS (Table 3, HR =1.32, 95% CI: 1.00–1.57, *P*=0.000).

**Table 2:** Association of TINCR and clinical factors with DFS

Clinicopathologic parameters	Hazard ratio	95% Confidence interval		*P*-value
		Lower limit	Upper limit	
Age (years) (<55 compared with ≥55)	0.81	0.52	1.29	0.321
Gender (male compared with female)	1.05	0.64	1.76	0.933
AFP (ng/ml) (<20 compared with ≥20)	1.98	1.77	2.34	0.002
HbsAg (absent compared with present)	1.03	0.56	1.82	0.931
Liver cirrhosis (absent compared with present)	1.15	0.75	1.79	0.568
Tumor size (cm) (<5 compared with ≥5)	1.93	1.64	2.36	0.001
TNM stage (I–II compared with III–IV)	2.26	1.27	3.88	0.004
Differentiation status (well/moderate)	2.27	1.24	4.65	0.036
Vascular invasion (absent compared with present)	1.93	1.59	2.28	0.000
TINCR expression (low compared with high)	1.82	1.28	2.55	0.001

**Table 3:** Multivariate analyses of clinicopathological and TINCR expression for DFS

Clinicopathologic parameters	Hazard Ratio	95% Confidence interval		*P*-value
		Lower limit	Upper limit	
Tumor size (cm) (<5 compared with ≥5)	1.38	1.04	1.71	0.010
AFP (ng/ml) (<20 compared with ≥20)	1.47	1.14	1.86	0.004
Vascular invasion (absent compared with present)	1.35	1.01	1.64	0.008
TINCR expression (low compared with high)	1.32	1.00	1.57	0.000

### Association of TINCR expression with OS of patients

**Figure 3:**
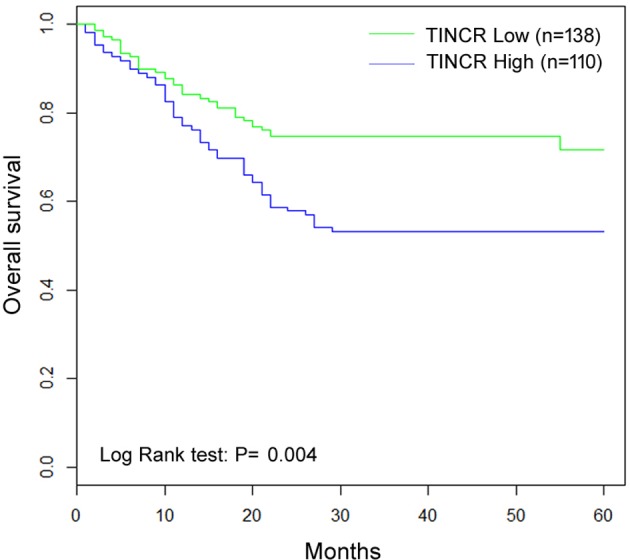
Kaplan–Meier curves for OS of HCC patients with high and low *TINCR* mRNA expression levels Patients with high *TINCR* mRNA levels (fold change <2) tended to have a shorter OS time (*P*=0.004).

As shown in Table 4, there was a statistically significant association between OS and TINCR expression level (*P*<0.01). Kaplan–Meier analysis showed that patients with HCC of high TINCR expression had worse OS compared with patients with tumor of low TINCR expression (Figure 3, log-rank test: *P*=0.004). At the 5-year follow-up, survived patients with low TINCR expression accounted for 77.8%, whereas survived patients with high TINCR expression accounted for 61.4%. In addition, differentiation status (log-rank test: *P*=0.005), AFP (log-rank test: *P*=0.010), tumor size (log-rank test: *P*=0.001), and TNM stage (log-rank test: *P*=0.000) were all identified to be prognostic factors for OS of patients with HCC. However, no prognostic value of gender, age, HbsAg, or liver cirrhosis was found on OS of HCC patients. Moreover, multivariate analysis showed TINCR as an independent prognostic factor for OS (Table 5, HR =1.57, 95% CI: 1.30–1.86, *P*=0.004).

**Table 4:** Association of TINCR and clinical factors with OS

Clinicopathologic parameters	Hazard ratio	95% Confidence interval		*P*-value
		Lower limit	Upper limit	
Age (years) (<55 compared with ≥55)	0.77	0.37	1.55	0.446
Gender (male compared with female)	1.00	0.45	2.23	1.000
AFP (ng/ml) (<20 compared with ≥20)	1.68	1.33	2.04	0.010
HbsAg (absent compared with present)	1.52	0.56	3.92	0.471
Liver cirrhosis (absent compared with present)	1.49	0.77	2.95	0.224
Tumor size (cm) (<5 compared with ≥5)	1.57	1.18	1.90	0.001
TNM stage (I–II compared with III–IV)	22.1	5.62	87.6	0.000
Differentiation status (well/moderate)	2.15	1.29	3.85	0.005
Vascular invasion (absent compared with present)	1.54	1.23	1.96	0.002
TINCR expression (low compared with high)	1.63	1.23	2.54	0.002

**Table 5:** Multivariate analyses of clinicopathological and TINCR expression for OS

Clinicopathologic parameters	Hazard ratio	95% Confidence interval		*P*-value
		Lower limit	Upper limit	
Tumor size (cm) (<5 compared with ≥5)	1.62	1.34	1.97	0.001
AFP (ng/ml) (<20 compared with ≥20)	1.71	1.47	2.09	0.000
TNM stage (I–II compared with III–IV)	1.18	0.92	1.49	0.224
Differentiation status (well/moderate)	1.17	0.96	1.33	0.236
Vascular invasion (absent compared with present)	1.46	1.14	1.77	0.000
TINCR expression (low compared with high)	1.57	1.30	1.86	0.004

### TINCR expression was inhibited by *miR-137* and *miR-133a*

**Figure 4:**
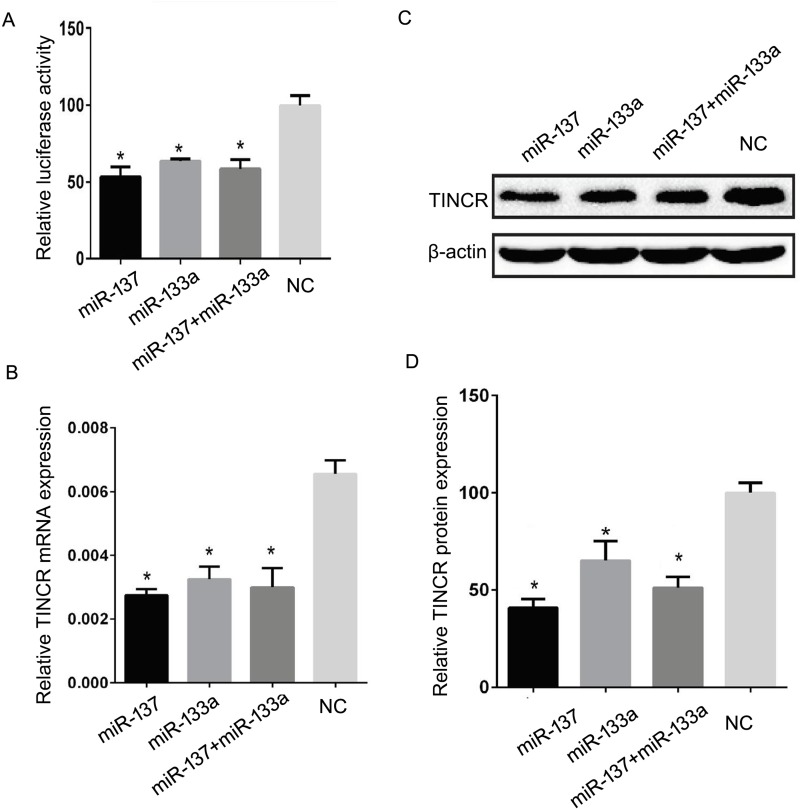
TINCR expression was inhibited by *miR-137*/*miR-133a* (**A**) *miR-137, miR-133a*, and *miR-137*/*miR-133a* cotransfection could suppress the luciferase activity in pmirGLO-TINCR transfected HUH7 cells. (**B**) Compared with negative control, *miR-137, miR-133a*, and *miR-137*/*miR-133a* cotransfection could significantly reduce the *TINCR* mRNA expression in HUH7 cells. (**C, D**) *miR-137, miR-133a*, and *miR-137*/*miR-133a* cotransfection led to dramatic reduction in TINCR protein expression in HUH7 cells. *P<0.05.

In order to investigate whether TINCR expression could be regulated by miRNAs in HCC, prediction algorithms, including miRWalk [16] and starBase [17] were used to screen the miRNAs that potentially target TINCR. Based on these data and the previous reports about the candidate miRNAs’ function, six cancer-related or tumor-suppressing miRNAs were chosen for further investigation, including *miR-198, miR-126, miR-133a, miR-137, miR-22*, and *miR-372* [18–23]. The luciferase assays demonstrated that *miR-137* and *miR-133a*, rather than *miR-126, miR-22*, and *miR-372* could significantly suppress the luciferase activity in pmirGLO-TINCR (3′-UTR) and miRNAs cotransfected cells (Figure 4). Data showed that *miR-137* and *miR-133a* transfection led to 45.14 ± 7.31% and 35.87 ± 3.24% decrease in luciferase activity in HUH7 cells, respectively (Figure 4A, *P*=0.002 and 0.006). We next detected whether *miR-137* and *miR-133a* could decrease *TINCR* mRNA expression levels in HCC cells. Results showed that *miR-137* and *miR-133a* could significantly reduce the *TINCR* mRNA expression level by 56.74 ± 4.92% and 50.13 ± 8.64% in HUH7 cells (Figure 4B, *P*=0.003 and 0.002), respectively. Moreover, the expression of TINCR protein was inhibited significantly in HUH7 cells by *miR-137* and *miR-133a*, with the TINCR protein level decreased to 58.96 ± 2.52% and 35.03 ± 8.85% (Figure 4C,D, *P*=0.001 and 0.017), respectively. These data suggest that TINCR is a direct target of *miR-137*/*miR-133a*, which is suppressed by *miR-137*/*miR-133a*.

To investigate whether *miR-137* and *miR-133a* could synergistically inhibit TINCR expression in HCC, the HUH7 cells were cotransfected with *miR-137* and *miR-133a*, and subjected to luciferase assay and TINCR expression detection. We found that *miR-137* and *miR-133a* co-transfection significantly decreased the luciferase activity (40.59 ± 6.37%), *TINCR* mRNA expression (53.06 ± 10.84%) and TINCR protein level (47.55± 5.08%) in HUH7 cells (Figure 4, *P*<0.05). However, *miR-137*/*miR-133a* cotransfection showed no significant changes when compared with *miR-137* or *miR-133a* alone (Figure 4, *P*> 0.05). Although *miR-137*/*miR-133a* cotransfection seemed to show more power than *miR-133a* in suppressing the expression of TINCR, it displayed less activity than *miR-137*. Therefore, we could not conclude that *miR-137* and *miR-133a* have synergistic roles in inhibiting TINCR expression in HCC.

## Discussion

HCC accounts for approximately 70–90% of primary liver carcinoma worldwide, becoming one of the top three major causes of cancer-related mortality [24]. Recently, novel strategies, such as gene therapy and targetted therapy have become available and have been developed for HCC treatment. However, the 5-year survival rate for patients with HCC is still very low [14]. Therefore, to develop novel and reliable biomarkers, it is necessary for evaluation of the prognosis and efficacy of the therapeutic strategies for HCC.

Recently, several literatures indicated that expressions of lncRNAs were significantly altered between HCC tissues and nontumor tissues by lncRNAs microarray technology interrogating putative lncRNAs. For example, Zhu et al. [25] suggested 174 lncRNAs were differentially expressed between five pairs of HCC samples and nontumor samples. Gao et al. [26] conducted the study that aberrant expression of lncRNAs might be responsible for the HCC invasion and metastasis. Zhang et al. [27] reported that LINC01419 was significantly up-regulated in HBV-related and HCV-related HCC when compared with matched nontumor liver tissues.

However, the prognostic implication of TINCR was rarely studied. Current reports showed that TINCR had remarkable impacts on cell proliferation, apoptosis, and angiogenesis promotion, therefore regarding as a biomarker which was significantly associated with cancer metastasis and unfavorable prognosis [28,29]. TINCR expression was significantly up-regulated in bladder cancer tissues and cells, showing strongly increased TINCR expression level in the higher tumor stage [30]. In human gastric carcinoma, up-regulated TINCR was found to contribute to oncogenesis and cancer progression through influencing cell proliferation and apoptosis [31]. In addition, aberrant TINCR expression has also been demonstrated in human squamous cell carcinoma [32,33]. In the current study, HCC patients with high TINCR expression had larger tumor size and advanced stage, suggesting that TINCR could be involved in the development and progression of HCC. Furthermore, RT-PCR results revealed that TINCR was significantly up-regulated in HCC. Up-regulation of TINCR expression has been demonstrated to remarkably correlate with tumor size, tumor differentiation, TNM stage and vascular invasion, which revealed that TINCR expression was correlated with advanced tumor progression and aggressive clinicopathological features. High TINCR expression served as an independent factor in unfavorable survival and worse outcomes in HCC patients. Our data suggest that TINCR expression could be a valuable marker of prognosis and malignant progression of glioma.

In the present study, lncRNA TINCR was identified as an independent factor for DFS and OS in a cohort of 248 patients with HCC. Patients with high TINCR expression had a shorter survial rate than those with low TINCR expression. These data demonstrated that TINCR was of clinical implication in prognosis of patient with HCC. As far as we know, this is the first study that proposes the clinical significance of TINCR expression in predicting the OS and RFS of patients with HCC. In our study, up-regulation of TINCR expression was closely associated with HCC progression. These findings may provide a new potential therapeutic biomarker for HCC diagnosis. However, further investigations are needed to illuminate the detailed molecular mechanism by which lncRNA TINCR plays a role in HCC.

## Abbreviations

AFP: α-fetoprotein
ALT: alanine transaminase
DFS: disease-free survival
HbsAg: hepatitis B surface antigen
HCC: hepatocellular carcinoma
lncRNA: long noncoding RNA
OS: overall survival
TBST: 0.1% TBS-Tween 20
TINCR: terminal differentiation induced ncRNA
TNM: Tumor Node Metastasis
HR: heart rate
95% CI: 95% Confidence interval
